# A Treatise on Military Surgery and Hygiene

**Published:** 1865-01

**Authors:** 


### Book Notices.

A Treatise on Military Surgery and Hygiene. By Frank Hastings Hamilton, M.D., late Lieutenant-Colonel, Medical Inspector U.S.A.; Professor of Military Surgery and Hygiene, and of Fractures and Dislocations in Bellevue Medical College; Surgeon to Bellevue Hospital; Professor of Military Surgery, &c., in Long Island College Hospital; author of "Treatise on Fractures and Dislocations," and of a "Practical Treatise on Military Surgery." Illustrated with 127 Engravings. New York: Bailliere Brothers, 520 Broadway.

This is a full-sized octavo volume of 648 pages, printed on fair type, good paper, and neatly bound.

The Author is one of the most careful observers and best writers in the profession. Combining ample experience in the field, the camp, and the hospital, with long study and discipline as a teacher in surgery, we should expect from his pen a work of the highest order of merit.

The general scope of the work will be best indicated by the following table of contents:—

*Chapter* 1. Introduction. *Chap.* 2. Examination of Recruits. *Chap.* 3. General Hygiene of Troops. *Chap.* 4. Bivouac, Accommodation of Troops in Tents, Barracks, Billets, Huts, &c. *Chap.* 5. Hospitals. *Chap.* 6. Preparations for the Field. *Chap.* 7. Hygienic Management of Troops upon the March. *Chap.* 8. Conveyance of Sick and Wounded Soldiers. *Chap.* 9. Gun-Shot Wounds. *Chap.* 10. Gun-Shot Injuries of the Head. *Chap.* 11. Gun-Shot Injuries of the Face and Neck. *Chap.* 12. Gun-Shot Injuries of the Thorax. *Chap.* 13. Fractured and Incised Wounds of the Thorax. *Chap.* 14. Gun-Shot Wounds of the Abdomen. *Chap.* 15. Punctured and Incised Wounds of the Abdomen. *Chap.* 16. Gun-Shot Wounds of the Male Organs of Generation. *Chap.* 17. Gun-Shot Fractures. *Chap.* 18. Amputations. *Chap.* 19. Exsections. *Chap.* 20. Arrow Wounds. *Chap.* 21. Traumatic Gangrene. *Chap.* 22. Hospital Gangrene. *Chap.* 23. Dry Gangrene. *Chap.* 24. Tetanus. *Chap.* 25. Scorbutus. *Chap.* 26. On the Employment of Anæsthetics in Major Amputations, and in other Severe Surgical Operations, after Gun-Shot Injuries. Appendix.

Such are the subjects treated in the work before us. From the brief examination we have been able to make, we do not hesitate to recommend it as the best work on Military Surgery and Hygihne, now accessible to the profession, in this country.

We would especially commend the Hygienic part of the work.

